# Molecular Dynamics Simulations of Acylpeptide Hydrolase Bound to Chlorpyrifosmethyl Oxon and Dichlorvos

**DOI:** 10.3390/ijms16036217

**Published:** 2015-03-18

**Authors:** Hanyong Jin, Zhenhuan Zhou, Dongmei Wang, Shanshan Guan, Weiwei Han

**Affiliations:** 1 Key Laboratory for Molecular Enzymology and Engineering of the Ministry of Education, College of Life Science, Jilin University, Changchun 130023, China; E-Mails: hyjin13@mails.jlu.edu.cn (H.J.); dmwang12@mails.jlu.edu.cn (D.W.); 2 Second Bethune Hospital of Jilin University, Changchun 130041, China; E-Mail: zhouzhenhuan1979@163.com; 3 State Key Laboratory of Theoretical and Computational Chemistry, Institute of Theoretical Chemistry, Jilin University, Changchun 130023, China; E-Mail: guanss12@mails.jlu.edu.cn

**Keywords:** acylpeptide hydrolase, organophosphorus compound, docking study, molecular dynamics simulation

## Abstract

Acylpeptide hydrolases (APHs) catalyze the removal of *N*-acylated amino acids from blocked peptides. Like other prolyloligopeptidase (POP) family members, APHs are believed to be important targets for drug design. To date, the binding pose of organophosphorus (OP) compounds of APH, as well as the different OP compounds binding and inducing conformational changes in two domains, namely, α/β hydrolase and β-propeller, remain poorly understood. We report a computational study of APH bound to chlorpyrifosmethyl oxon and dichlorvos. In our docking study, Val471 and Gly368 are important residues for chlorpyrifosmethyl oxon and dichlorvos binding. Molecular dynamics simulations were also performed to explore the conformational changes between the chlorpyrifosmethyl oxon and dichlorvos bound to APH, which indicated that the structural feature of chlorpyrifosmethyl oxon binding in APH permitted partial opening of the β-propeller fold and allowed the chlorpyrifosmethyl oxon to easily enter the catalytic site. These results may facilitate the design of APH-targeting drugs with improved efficacy.

## 1. Introduction

Acylaminoacyl peptidase (APH) from hyperthermophilic *Aeropyrum pernix* K1 belongs to prolyloligopeptidase (POP; EC 3.4.21.26) family of serine proteases; this family also includes dipeptidyl peptidase IV (EC 3.4.14.5) and oligopeptidase B (OB; EC 3.4.21.83); APH also catalyzes *N*-terminal hydrolysis of *N*_α_-acylpeptides to release *N*_α_-acylated amino acids [1,2,3,4,5,6,7,8,9]. POP family members consist of two domains, namely, α/β hydrolase and β-propeller; classic serine proteases exhibit radically different β/β (chymotrypsin) and α/α (subtilisin) protein scaffolds, although these proteases show similar catalytic triads [9]. Like other POP family members, APHs are also believed to be important targets for drug design. For example, while human APH is known to be deficient in small-cell lung and renal carcinomas, a role in the malignant state of these cell lines has not so far been established [10,11,12]. Furthermore, porcine brain APH is potently inhibited by organophosphorous compounds and has been proposed as a new pharmacological target for the cognitive-enhancing effects of these compounds in the treatment of neurodegenerative diseases [7,10].

**Figure 1:**
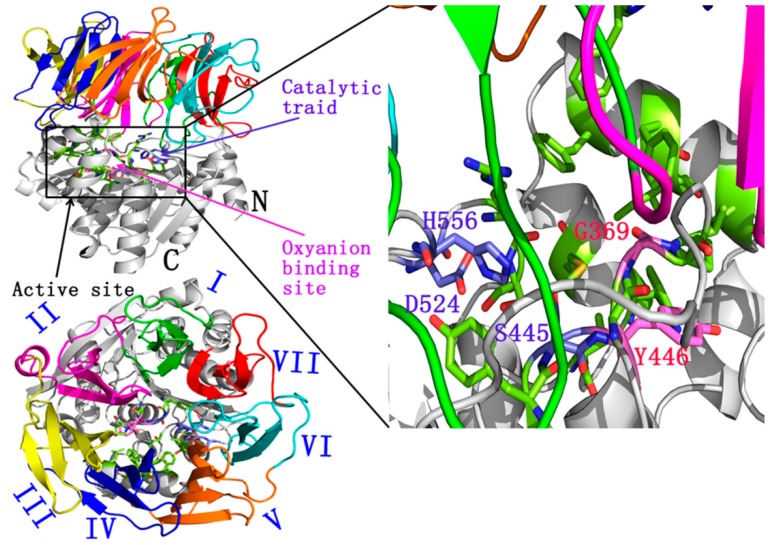
The active triad of acylpeptide hydrolases (APH): Ser445, His556, and Asp524. Ser445, Gly369 and Tyr446 function as oxyanion hole. PDB Id (1VE7).

The crystal structure of apAPH, which is the first available APH structure, was determined in 2004 (PDB Id 1VE7) [9]. This enzyme is active and very stable at an optimal temperature of 90 °C. The structure of apAPH is a symmetric homodimer, in which each subunit comprises two domains. The *N*-terminal domain (residues 24–324) is a propeller with seven blades; each blade consists of a four-stranded antiparallel β sheet. The main residues 325–581 exhibit a canonical α/β hydrolase fold, with a central eight-strand mixed β sheet flanked by five helices on one side and six helices on the other side. A short α-helix at the *N*-terminal (residues 8–23) extends from the β-propeller domain and forms a part of the hydrolase domain. Ser445, His556, and Asp524 constitute a catalytic triad; Ser445, Tyr446 and Gly369 function as an oxyanion hole (Figure 1) [9]. 

Serine hydrolases can react with organophosphorus (OP) compounds [13,14,15]. In 2000, porcine APH was reported as potently inhibited by OP compounds (chlorpyrifos methyl oxon and dichlorvos; IC_50_ values of 18.3 and 118.7 nM for 20 min, respectively) [7]. The *in vitro* sensitivity of APH to these compounds ranges between six and ten times greater than that of acetylcholinesterase (AChE); thus, APH is a target of pharmacological and toxicological significance [7].

To date, many experimental [3,5,16] and theoretical studies [17,18,19] have focused on the relationship between substrates and APH. However, no theoretical studies have reported the relationship between OP and APH. 

The propensity of small molecule binding to macromolecules regulates their bioavailability and subcellular disposition [20]. Molecular dynamics (MD) simulations are able to provide information about mechanical properties as well as structural changes within proteins, or in protein–protein and protein–ligand (substrate or inhibitor) complexes [21,22]. Thus, MD simulation has been widely used and the results are often able to reproduce results from experimental data and to be useful as a predictive tool in drug design by providing binding affinity estimates [21,22]. 

The binding free energy is also an important thermodynamic property that may be predicted in computational modeling of biological systems [23,24]. Recently, there have been an increasing number of studies that have attempted to predict the binding free energy of small molecules to proteins in combination with experimental measurements. The methods for estimating binding free energies include molecular mechanics/Poisson–Boltzmann surface area (MM/PBSA) [25,26], free energy perturbation (FEP) [23] linear interaction energy (LIE) [27], metadynamics [28], replica exchange umbrella sampling (REUS) [29], or umbrella sampling (US) [30]. Although the most predictive methods tend to use explicit solvent and can provide accurate prediction of binding affinities [31,32], they are often computationally expensive.

In this study, to characterize the different contributions of chlorpyrifosmethyl oxon and dichlorvos to APH activity, we performed molecular dynamics (MD) simulations and MM/PBSA calculations of APH and two inhibitors.

Despite advances in understanding the biological functions of acylpeptide hydrolases, little is known of the structural basis for the sequential deacetylation of *N*-terminally acetylated proteins. Until known, only the crystal of an APH from the thermophilic archaeon *Aeropyrum pernix* K1 (APH) was obtained for structure determination (PDB Id 1VE7) [9]. Although APH from *Aeropyrum pernix* K1 shares only 29%, 20% and 29% sequence identity with human, porcine, and rat acylpeptide hydrolase, respectively, there is a surprising conservation of secondary structure between mammalian APH and APH from *Aeropyrum pernix* K1, especially in the C-terminal domain (residues 325–581) having a canonical α/β hydrolase. It is well known that α/β hydrolase superfamily members have low sequence identity but similar function [33]. Thus, the theoretical study on APH with chlorpyrifosmethyl oxon and dichlorvos will provide a structural basis for the design of specific inhibitors for acylpeptide hydrolases.

## 2. Results and Discussion

### 2.1. Docking Study

**Figure 2:**
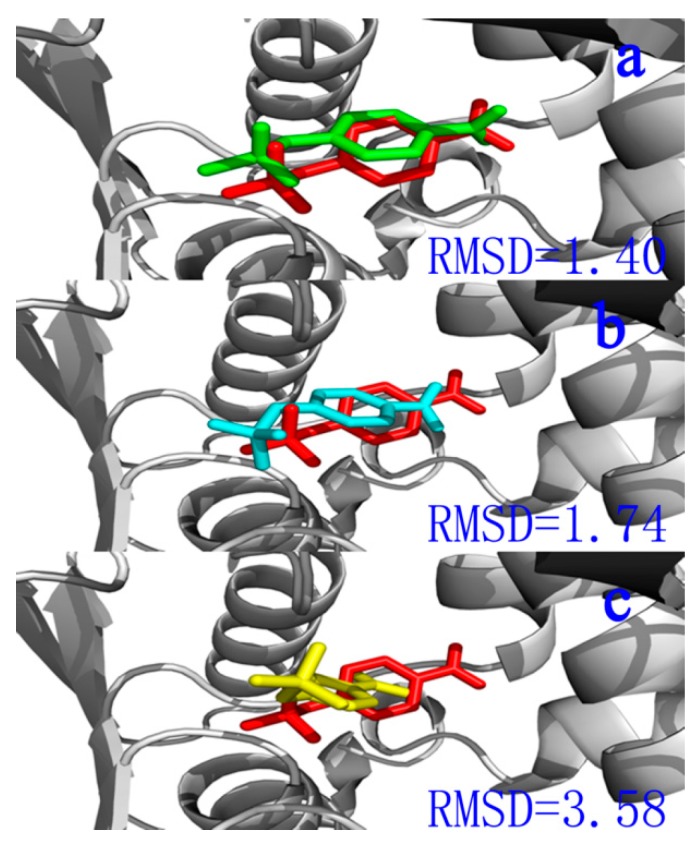
(**a**) The compartment between the docked ligand (green) and the reference for the crystal structure (red) located in the active site. Calculated by Autodock 4.2; (**b**) The compartment between the docked ligand (light blue) and the reference for the crystal structure (red) located in the active site. Calculated by Autodock vina; and (**c**) The compartment between the docked ligand (yellow) and the reference for the crystal structure (red) located in the active site. Calculated by CDOCKER.

Accurate generation and scoring of known ligand binding poses by a given procedure should be investigated [34,35]. Docking success is generally observed when the top scoring pose was approximately 2.0 to 2.5 Å heavy atom root-mean-square deviation (RMSD) of the crystal ligand [34,35,36]. A top-scoring pose not within 2.5 Å is defined as a scoring failure [36]. Figure 2a–c shows a representative example for a ligand (inhibitor) docked to a target, APH, with AutoDockVina [37], AutoDock 4.2 [38], and CDOCKER software [39]. The docked ligands were in the same orientation in different binding modes (Figure 2a–c). Compared with crystallographic reference, the ligand docked by AutoDock 4.2 [38] was successful (RMSD 1.40 Å). Thus, AutoDock 4.2 [38] was used for further docking analysis. 

**Figure 3:**
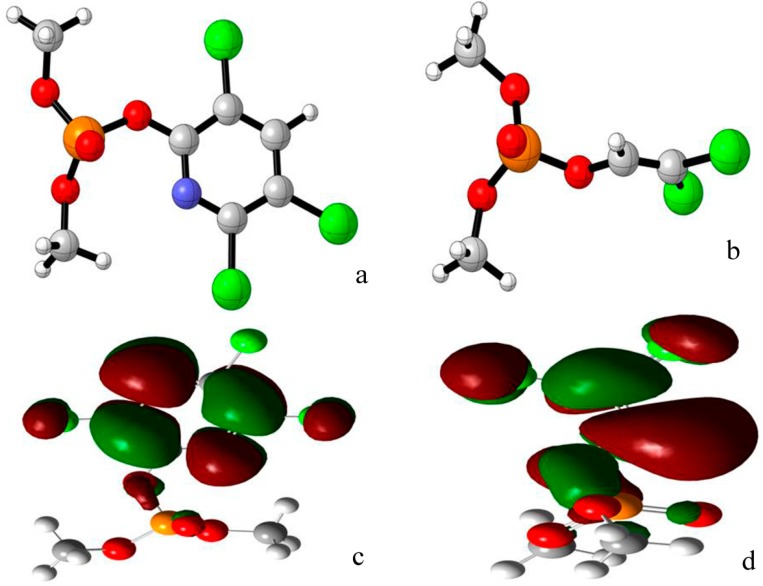
Chemical structure of (**a**) Chlorpyrifosmethyl oxon; (**b**) Dichlorvos generated by CLY view v1.0.561 beta; (**c**) The LUMO orbit of chlorpyrifosmethyl oxon; and (**d**) The LUMO orbit of dichlorvos generated by Gaussian View 5.0.

**Figure 4:**
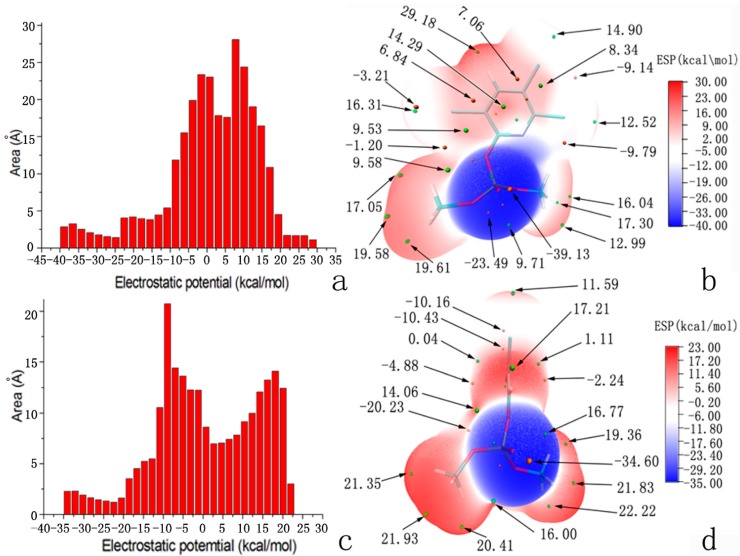
(**a**) Surface area in each electrostatic potential (ESP) range on the vdW surface of chlorpyrifosmethyl oxon; (**b**) ESP-mapped molecular vdW surface of chlorpyrifosmethyl oxon; (**c**) Surface area in each ESP range on the vdW surface of dichlorvos; and (**d**) ESP-mapped molecular vdW surface of dichlorvos. The unit is in kcal·mol^−1^.

The starting model of APH has been derived from 2.7 Å resolution crystal structure of a protein (PDB code 1VE7) [9]. The 3D structures of chlorpyrifosmethyl oxon and dichlorvos were download from ChemSpider database (Figure 3a,b) and optimized at the B3LYP-6-31G* level by using Gaussian 09 software [38]. The lowest unoccupied molecular orbital (LUMO) orbit of chlorpyrifosmethyl oxon and dichlorvos was generated by Gaussian view 5.07 (Figure 3c,d) [40], which indicated that the chlorine substituent group was the active center of the two inhibitors. Electrostatic potential (ESP) on molecular vdW surface is necessary to investigate and predict intermolecular interactions. In-depth investigation of the ESP of chlorpyrifosmethyl oxon (Figure 4b) and dichlorvos (Figure 4d) provides further insights into important interactions between OP compounds and APH. The ESP-mapped vdW surface along with surface extrema of chlorpyrifosmethyl oxon and dichlorvos is shown in Figure 4a,c, and the surface area in different ESP ranges was plotted using Multiwfn program (Figure 4b,) [41]. The values were 29.2 and 39.1 (kcal·mol^−1^) for chlorpyrifosmethyl oxon and 22.2 and 34.6 (kcal·mol^−1^) for dichlorvos; their remarkable differences suggested that the ESP distribution on the vdW surface fluctuates more remarkably in chlorpyrifosmethyl oxon than in dichlorvos. The ESP distribution of chlorpyrifosmethyl oxon also covers greater scope than that of dichlorvos, which is a direct consequence of the large polarity of benzene groups. The O atom of the phosphate group of chlorpyrifosmethyl oxon shows a more negative charge than that of dichlorvos and is useful for the attack of the hydroxyl group of Ser445. 

**Figure 5:**
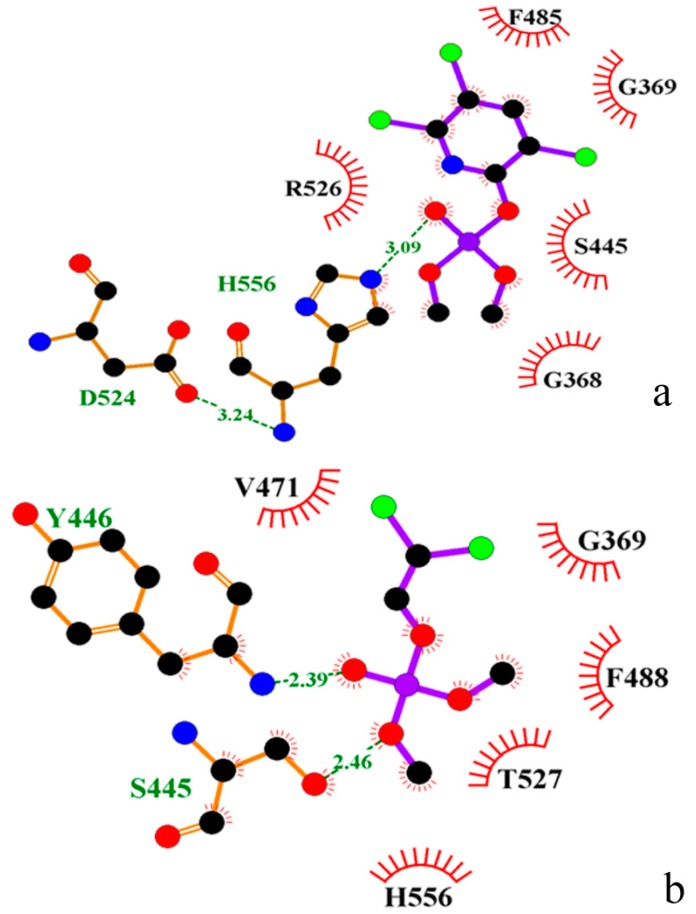
(**a**) chlorpyrifosmethyl oxon in the active pocket of APH; and (**b**) dichlorvos in the active pocket of APH drawn by LIGPLOT.

The two enzyme-inhibitor complexes were generated using AutoDock 4.2 [37]. The chlorpyrifosmethyl oxon and dichlorvos located at the active pocket. Figure 5 was drawn by LIGPLOT [42], in which the interactions shown are those mediated by hydrogen bonds and by hydrophobic contacts. From Figure 5, hydrogen bonds are indicated by dashed lines between the atoms involved, while hydrophobic contacts are represented by an arc with spokes radiating towards the ligand atoms they contact, and the contacted atoms are shown with spokes radiating back. From Figure 5a, His556 formed a weak hydrogen bond (3.09 Å) with chlorpyrifosmethyl oxon. In addition, Asp524 made a weak hydrogen bond (3.24 Å) with His556, helpful for Ser445 to attack the P atom of the chlorpyrifosmethyl oxon. Arg526, Phe485, Gly369, Ser445, and Gly368 made hydrophobic contacts with the chlorpyrifosmethyl oxon. From Figure 5b, Tyr446 and Ser445 formed two hydrogen bonds with dichlorvos. Val471, Gly369, Phe488, Thr527, and His556 made hydrophobic contacts with dichlorvos. However, in the APH-dichlorvos complex, there was no hydrogen bond between Asp524 and His556, which would have been useful for Ser445 to attack. 

Seen from Table 1, Arg526, Gly369, Ser445, His556, Val471, Asp524 and Gly368 have the highest conservation in hyperthermophilic *Aeropyrum pernix* K1, human, porcine, and rat APH, suggesting that these residues may play a crucial role in substrate recognition and/or transition state stabilization. Ser445, His556, and Asp524 functioned as a catalytic triad, and Gly369 acted as an oxyanion binding site residue. Arg526 is an important residue for substrate recognition [9]. Our results are consistent with experimental data. In our docking study, Val471 and Gly368 are important residues for chlorpyrifosmethyl oxon and dichlorvos binding. 

**Table 1:** Sequence alignment of amino acids around Arg526 for major representatives of APH.

Enzyme	Residue Number ^a^										
	526	369	527	445	488	485	556	446	471	368	524
*A. pernix*	Arg	Gly	Thr	Ser	Phe	Phe	His	Tyr	Val	Gly	Asp
Human	Arg	Gly	Val	Ser	Cys	Phe	His	His	Val	Gly	Asp
Pig	Arg	Gly	Val	Ser	Ser	Phe	His	His	Val	Gly	Asp
Rat	Arg	Gly	Val	Ser	Leu	Leu	His	His	Val	Gly	Asp

^a^ Residue numbering according to APH.

### 2.2. Structural Stability in Conventional MD Simulations

**Figure 6:**
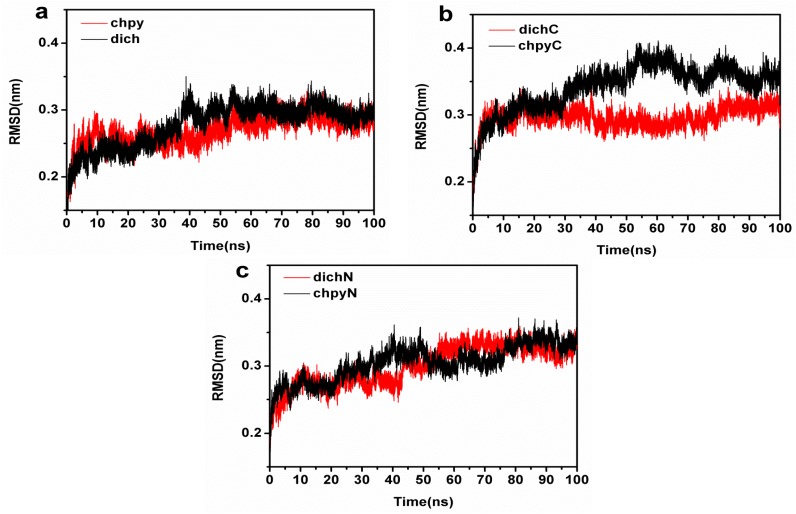
(**a**) Root-mean-square deviation (RMSD) plot of chlorpyrifosmethyl oxon (red) and dichlorvos (black) during 100 ns molecular dynamics (MD); (**b**) RMSD plot of α**/**β hydrolase domain (residues 8–23, 325–581) of chlorpyrifosmethyl oxon (black) and dichlorvos (red); and (**c**) RMSD plot of β-propeller domain (residues 24–324) of chlorpyrifosmethyl oxon (black) and dichlorvos (red).

Atom positional RMSD was calculated from MD simulations for the APH-chlorpyrifosmethyl oxon and APH-dichlorvos (Figure 6a). The RMSD for the APH-chlorpyrifosmethyl oxon stabilized about 0.28 nm around 60 ns, whereas the APH-dichlorvos reached a plateau at 0.32 nm for 50 ns. These results indicated that the protein structure spontaneously underwent significant conformational changes when the two inhibitors were docked during simulation. A detailed analysis of the simulation showed a particularly different high mobility of APH subdomains (the α/β hydrolase fold domain and β-propeller domain) between chlorpyrifosmethyl oxon and dichlorvos binding (Figure 6b,c). α/β Hydrolase fold domain varied differently in chlorpyrifosmethyl oxon and dichlorvos binding (Figure 6b). The backbone RMSD of α/β hydrolase fold domain in the chlorpyrifosmethyl oxon significantly increased by 0.4 nm for 50 ns to reach a short plateau and changed intensively; as a result, a larger flexibility is observed in APH-chlorpyrifosmethyl oxon complex than in APH-dichlorvos. However, the β-propeller domain varied similarly to chlorpyrifosmethyl oxon and dichlorvos binding. Chlorpyrifosmethyl oxon increased flexibility in the α/β hydrolase fold domain probably because of the benzene group that can produce π–π conjugation in the hydrophobic active pocket and can cause the conformational change in the α/β hydrolase fold domain.

**Figure 7:**
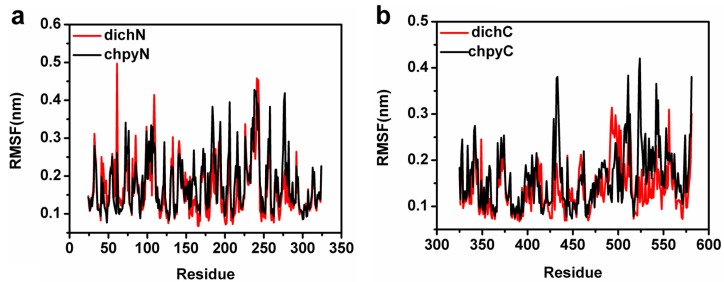
(**a**) RMSF plot during 100 ns MD (residues 24–324 (β–propeller domain)). Color black represent for chlorpyrifosmethyl oxon, and color red represents for dichlorvos; and (**b**) RMSF plot during 100 ns MD (α**/**β hydrolase domain (residues 325–581)). Color black represent for chlorpyrifosmethyl oxon, and color red represents for dichlorvos.

Atom-positional root-mean-square fluctuations (RMSFs) calculated for backbone atoms in the chlorpyrifosmethyl oxon-APH and dichlorvos-APH trajectories with respect to the initial conformations were the MD-final conformations used to compare regions, in which the dynamics differed among these systems (Figure 7a,b). Seen from Figure 7a, the residues (61, 99, 235–244) that contribute mostly to the motions in the simulations were β–propeller domain; these residues in turn contribute significantly to the motion with dichlorvos binding. The large atomic fluctuations observed in the chlorpyrifosmethyl oxon-bound ensemble were located in the α/β hydrolase fold domain (residues 432–434, 511, 542, and 581) (Figure 7b). These collective motions resulted in significant conformational for chlorpyrifosmethyl oxon binding in APH which can produce π–π conjugation in the hydrophobic active pocket and cause conformational change in the α/β hydrolase fold domain. The propeller blades acted as a gating filter during catalysis by mutation analysis of POP [43,44]. This structural feature of chlorpyrifosmethyl oxon binding in APH permits partial opening of the β-propeller fold and allows chlorpyrifosmethyl oxon to easily enter the catalytic site. 

Hydrogen-bond occupancies were analyzed for residues between the α/β hydrolase fold domain and β-propeller domain in two simulations (Table 2). There are more twenty hydrogen bonds, and salt bridges were found between the α/β hydrolase fold domain and β-propeller domain. During MD simulations, only nine hydrogen bonds appeared between the α/β hydrolase fold domain and the β-propeller domain (Table 2). Except for two hydrogen bonds (Leu302–Asp376 and Arg327–Pro323) located at the edge of the α/β hydrolase fold domain and the β-propeller domain, other hydrogen-bond occupancies were higher in the dichlorvos-APH than in the chlorpyrifosmethyl oxon-APH. The lower hydrogen bond occupancy indicated that the β-propeller domain moved away from the catalytic domain and allowed the chlorpyrifosmethyl oxon to easily enter the catalytic site.

**Table 2:** Hydrogen bonds occupancies for the α/β hydrolase fold domain and β-propeller domain with chorpyrifosmethyl oxon and dichlorvos bound APH during MD simulations.

Hydrogen Bonds	Distance (Å)	Chorpyrifosmethyl Oxon-APH	Dichlorvos-APH
VAL46:HN-ASN559:O	2.41	<10	0.19
ARG113:HH22-SER525:O	2.43	<10	0.12
GLY173:HN-GLN491:OE1	1.63	<10	0.26
ASN284:HD21-ASP376:O	1.64	0.39	0.77
LEU302:HN-ASP376:OD1	2.13	0.93	0.94
ARG327:HN-PRO323:O	1.91	0.91	0.92
GLU405:HN-THR214:O	1.98	<10	0.45
ARG408:HH22-GLY173:O	1.88	<10	0.15

**Figure 8:**
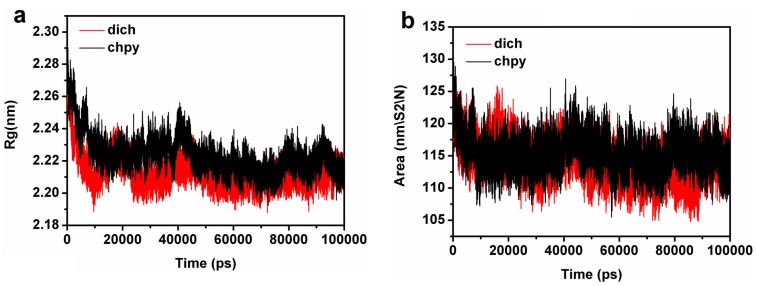
(**a**) Radius of gyration (Rg) for the chlorpyrifosmethyl oxon (black) and dichlorvos (red) bound to APH; and (**b**) Solvent accessible surface area for the chlorpyrifosmethyl oxon (black) and dichlorvos (red) bound to APH.

Radius of gyration (R_g_) refers to several related measures of the size of an object, surface, or ensemble of points. R_g_ is calculated as the root mean square distance of the object parts from either the center of gravity or a given axis. R_g_ of the protein is represented by protein volume and shape. Figure 8a shows R_g_ of APH-chlorpyrifosmethyl oxon (black) and APH-dichlorvos (red). The mean R_g_ of APH-chlorpyrifosmethyl oxon was 2.24 nm, whereas the mean R_g_ of APH-dichlorvos was 2.22 nm. Furthermore, the mean R_g_ for the APH-chlorpyrifosmethyl oxon was larger than that of the APH-dichlorvos complex. This finding may facilitate the conformational rearrangement of the β-propeller domain to move away from the catalytic domain in the APH-chlorpyrifosmethyl oxon complex.

Time-dependent solvent-accessible surface area was also calculated for the structural assembly from the simulations (Figure 8b). After a period of 100 ns, the APH-chlorpyrifosmethyl oxon complex became larger than that of the APH-dichlorvos complex. In the larger active site, the nucleophilic OH^−^ can be easily attached to the ligand. 

### 2.3. Principal Component Analysis and Free-Energy Landscape

**Figure 9:**
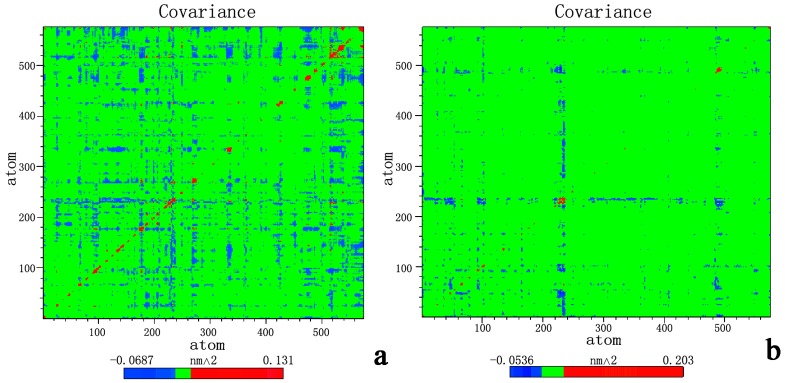
Cross-correlation matrix of the fluctuations of each of the *x*, *y*, and *z* coordinates of the Cα atoms from their average during 100 ns MD (**a**) chlorpyrifosmethyl oxon; and (**b**) dichlorvos. Blue color represents the negative anticorrelation, green represents noncorrelated, random motions, and red represents positive correlation. The two figures were made using Adobe Illustrator CS5.

The cross-correlations of the Cα atomic displacements of chlorpyrifosmethyl oxon and dichlorvos bound APH are illustrated in Figure 9a,b, respectively. Highly positive regions (blue) indicate strong correlation in the movement of specific residues, whereas negative regions (red) are associated with strong anticorrelated motion of the residues. The same principle is applied to highly anticorrelated motions (red), in which very few motions occur. As expected, no large-scale conformational change occurred during the timescale of our simulations. In the case of chlorpyrifosmethyl oxon-APH (Figure 9a), the strongest correlated motions occurred in the α/β hydrolase fold domain and β-propeller domain; by contrast, the presence of dichlorvos in the dichlorvos-APH (Figure 9b) significantly decreased the correlated motions in the α/β hydrolase fold domain and β-propeller domain. Cross-correlation analysis revealed a complex pattern of correlated and anticorrelated motions in the α/β hydrolase fold domain and β-propeller domain motions; these motions were significantly more affected by the presence of the chlorpyrifosmethyl oxon than by dichlorvos.

**Figure 10:**
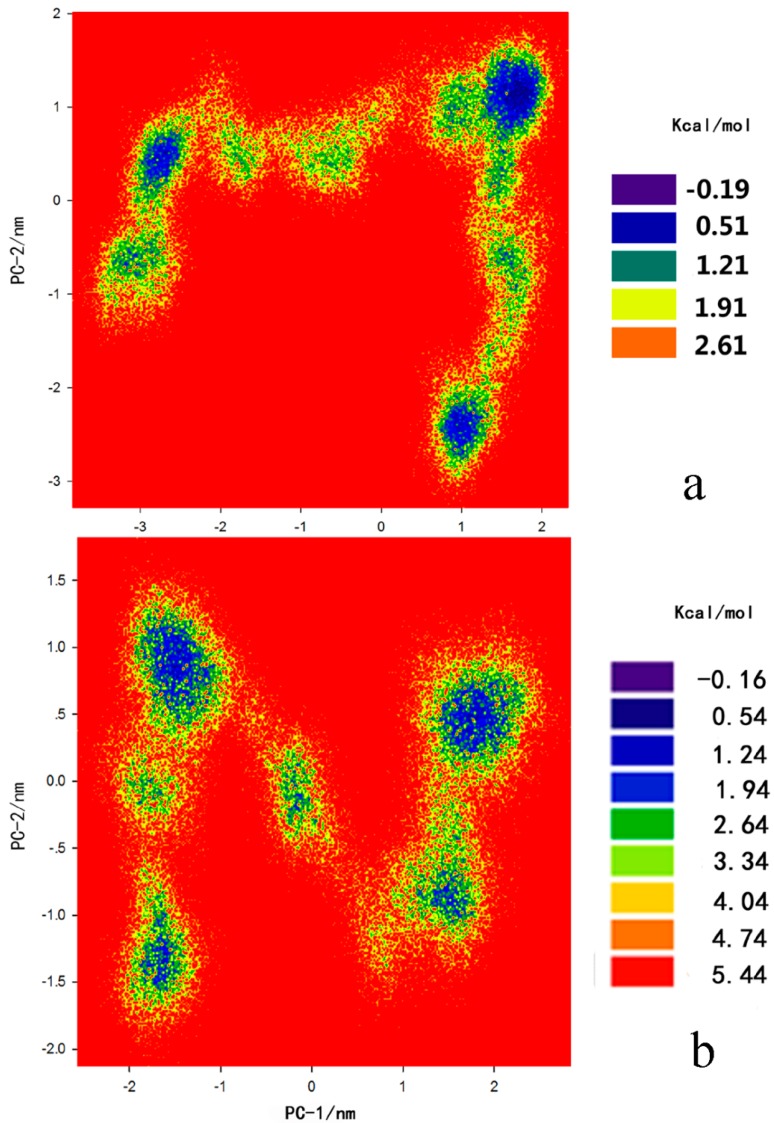
The relative Free energy surfaces along the first two principle components (PC-1, PC-2) of (**a**) chlorpyrifosmethyl oxon-APH; and (**b**) dichlorvos-APH during 100 ns generated by Sigma plot 12.0 (12.0, Systat software company, San Jose, CA, USA).

To further inspect the direction of the fluctuation in the two systems, we performed the free energy landscape (FEL) for all Cα atoms of the protein-inhibitor complex structure from 100 ns trajectory. Figure 10a,b display the corresponding free energy contour map with deeper blue color indicating lower energy (Δ*F* = −*RT* × ln *P*, where *P* is the relative probability in a region). A lower relative free energy of the complex indicated a stronger conformational stability of the complex. The lowest relative free energy between the chlorpyrifosmethyl oxon and APH (−0.19 kcal·mol^−1^) was lower than that of the dichlorvos-APH (−0.16 kcal·mol^−1^) (Figure 10a). The conformations of chlorpyrifosmethyl oxon-APH are also distributed more compactly than the dichlorvos-APH complex; this result indicated that APH is mostly affected by the presence of chlorpyrifosmethyl oxon.

### 2.4. MM/PBSA Calculation 

The binding free energy from MM/PBSA methodology can provide a semi-quantitative estimate of substrate (inhibitor) affinity with enzyme. Table 3 shows the binding free energies and their components for the two inhibitors. The binding free energies (∆*G*_bind_) of the two inhibitors were both negative values, indicating that these inhibitors were energetically favorable (Table 3). The two binding free energies were also compared, and the results showed that the chlorpyrifosmethyl oxon was lower in energy than APH with dichlorvos. This result suggested that the chlorpyrifosmethyl oxon has a highly probable binding energy. Our results were consistent with the experimental data [7]. For each component of MM/PBSA binding free energies, vdW energies (∆*E*_vdW_) contribute to total energies to a greater extent than electrostatic energies (∆*E*_ele_) in the two inhibitor-APH complexex. Hence, vdW interaction was observed in the dominant position in the interaction of the two inhibitors with APH. These results are consistent with the observations in MD trajectories. Furthermore, they reveal that the binding mode of APH-chlorpyrifosmethyl oxon is an optimal enzyme complex conformation.

**Table 3:** The MM-PBSA score for the two complexes (kcal·mol^−1^).

Energy Components (kcal·mol^−1^)	Dichlorvos	Chlorpyrifosmethyl Oxon
∆***E*_ele_**	−18.28	−2.63
∆***E*_vdW_**	−28.49	−29.64
∆***G*_PB_^a^**	31.60	15.09
∆***G*_np_^b^**	−4.08	−3.72
**Nonpolar**	−32.57	−33.36
**Polar**	13.32	12.46
∆***G*_bind_**	−19.25	−30.90

^a^ the solvation energy of polar part; ^b^ the solvation energy of nonpolar part.

## 3. Experimental Section 

### 3.1. Docking Study

AutoDock 4.2 [38], AutoDock Vina [40], and CDOCKER of Discovery Studio 2.5 (Accelrys Inc., San Diego, CA, USA) [39] were used for docking. 

Lamarckian genetic algorithm was implemented in AutoDock 4.2 program suite [38] to identify appropriate binding modes and conformation of the ligand molecules. In all of the cases, grid maps with a box size of 48 Å × 48 Å × 48 Å points and grid-point spacing of 0.375 Å were used. Lamarckian genetic algorithm and pseudo-Solis and Wets method were applied for minimization by using default parameters. Ser445, His556, Asp524, andTyr446 were selected as flexible residues, and population size of 150 was set as the parameter. Simulations were performed using up to 2.5 million energy evaluations with a maximum of 27,000 generations. Each simulation was performed 10 times, yielding 10 docked conformations. The lowest energy conformations were regarded as the binding conformations between the ligands and the proteins. 

AutoDock Vina was used to docking study [37]. Default parameters were used as described in the manual of Vina unless otherwise specified.

Docking analysis was performed using Discovery Studio 2.5 software with fully automated docking tool in “Dockligands (CDOCKER)” [39]. CharmM was the force field applied to the receptor, and hydrogen was minimized. Force fields are applied on the molecules and minimized to obtain the lowest energy minimum structure. The generated initial structures of the ligand were further refined by simulated annealing. The CDOCKER energy of the most feasible poses docked into the receptor was calculated and compared with that of interacting residues in the active site region with a crystallized inhibitor in the APH.

### 3.2. Conventional Molecular Dynamics Simulations 

Simulations were performed using a CHARMM (Chemistry at HARvard Macromolecular Mechanics) force-field named charmm22* [45,46] with GROMACS 4.5 software (Herman Berendsen, Holland, The Netherlands). The systems were placed in a cubic box (proteins were placed at least 0.8 nm from the box edge), treated under periodic boundary conditions, and solvated with explicit SPC216 (simple point charge) model water molecules. The systems were neutralized with sodion (Na^+^) counterions as necessary. Before MD simulations were performed, the systems were energy minimized by the steepest descent algorithm to avoid any steric conflicts generated during the initial setup. NVT (Canonical ensemble) and NPT (isothermal-isobaric ensemble) equilibration of 500 ps each were performed to help the system reach the desired temperature and pressure. Bond lengths and angles were constrained using P-LINCS algorithm [47], and the geometry of water molecules was constrained by SETTLE algorithm [48]. A twin-range cutoff of1.2 nm was used for van der Waals (vdW) interactions, and long-range electrostatic interactions were treated by particle mesh Ewald method [30]. The equilibration procedure consisted of thermalization of the solvent, with the solute atoms fixed, for 500 ps at 353 K, followed by minimization of all solute atoms, keeping the solvent coordinates fixed, and simulation of the complete system by increasing the temperature from 0 to 353 K in 500 ps increments of 50 K each for MD simulations. Data were produced for 100 ns. System configurations were recorded as trajectory files for every 1.0 ps. For the ligands (chlorpyrifosmethyl oxon and dichlorvos), Dundee PRODRG [49] server was used to build a GROMACS topology for the two inhibitors. The .itp file (topology file of inhibitors) was added in the protein top file, and the two protein-inhibitor complexes were performed again using the CHARMM force-field named charmm22* [43,50]. 

### 3.3. Principal Component Analysis and Free-Energy Landscape

Principal component analysis (PCA) is a widely used approach for extracting the slow and functional motions of biomolecules from MD trajectories by applying dimensional reduction method [51]. PCA is based on the calculation and diagonalization of the covariance matrix (*C_ij_*) of the fluctuations of each of the *x*, *y*, and *z* coordinates of the Cα atoms (*N* = 581) from their average with 100 ns of the simulations for two models. For the displacement vectors ∆*r_i_* and ∆*r_j_* of atoms *i* and *j*, *C_ij_* is calculated as follows:

(1) $$C_{ij}=\frac{\langle ∆r_{i}\cdot ∆r_{j}\rangle }{{\left(\langle ∆r_{i}^{2}\rangle \cdot \;\langle ∆r_{j}^{2}\rangle \right)}^{1/2}}$$

where ∆*r_i_* (∆*r_j_*) is the displacement vector corresponding to the *i*th (*j*th) atom of the systems. The eigenvectors of the matrix are also called principal components (PCs), which represent the directions of the concerted motions. The first few PCs describe the slow-motion modes of the system; these modes are related to the functional motions of a biomolecular system [51]. The eigenvalues of the matrix indicates the magnitude of the motions along the direction. In this study, PCA was performed using GROMACS 4.5 to investigate and compare the modes of motion of the two systems. Free-energy landscape (FEL) can help elucidate dynamic processes in biological systems [52,53]. In FEL, the free-energy minima usually represent the conformational ensemble in stable states while the free energy barriers denote the transient states [54]. FEL was constructed based on the above PCA data. The corresponding expression is described as follows:

∆*G*(*X*) = −*K*_B_*T* ln *P*(*X*) (2)

where the reaction coordinate *X* is the PC, KB is the Boltzmann constant, *T* is the absolute temperature, and *P*(*X*) is the probability distribution of the system along the PC. In this study, FEL was calculated to identify the dominant conformational states of the two systems. The 3D FEL was generated by SigmaPlot 12.0.

### 3.4. Calculations of MM/PBSA Binding Free Energy

The lowest energy of the two structures with the last conformation at 100 ns MD simulations was used as a starting point to calculate binding free energies. Simulations were performed with Amber 11 package (Kollman, Los Angeles, CA, USA) for 10 ns by using the amber99sb force field parameter [46,55]. The binding free energies were calculated using molecular mechanics–Poisson–Boltzmann surface area (MM–PBSA) method [46].

Normal-mode analysis (NMA) is performed to estimate changes in solute entropy during ligand binding. However, NMA calculation is problematic and time-consuming; this approach does not consider solvent entropy. The two inhibitors used in the present study are also very similar. Therefore, the solute entropy term was disregarded. For each MD-simulated complex, Δ*G*_bind_ of the 1000 snapshots of the MD trajectory (one snapshot for each 2 ps during the last 2000 ps of the stable trajectory) and the final Δ*G*_bind_ were the average of calculated Δ*G*_bind_ for these snapshots.

## 4. Conclusions

APH from hyperthermophilic *Aeropyrum pernix* K1 belongs to the prolyl oligopeptidase family of serine proteases. APH catalyzes the *N*-terminal hydrolysis of *N*_α_-acylpeptides to release *N*_α_-acylated amino acids. To characterize the different contributions of chlorpyrifosmethyl oxon and dichlorvos to APH activity, we performed MD simulations of APH and the two inhibitors. These simulations indicate that two inhibitors induced different conformational changes in the α/β hydrolase fold domain containing an active triad and a β-propeller domain. Our study will help facilitate further studies regarding this topic.
